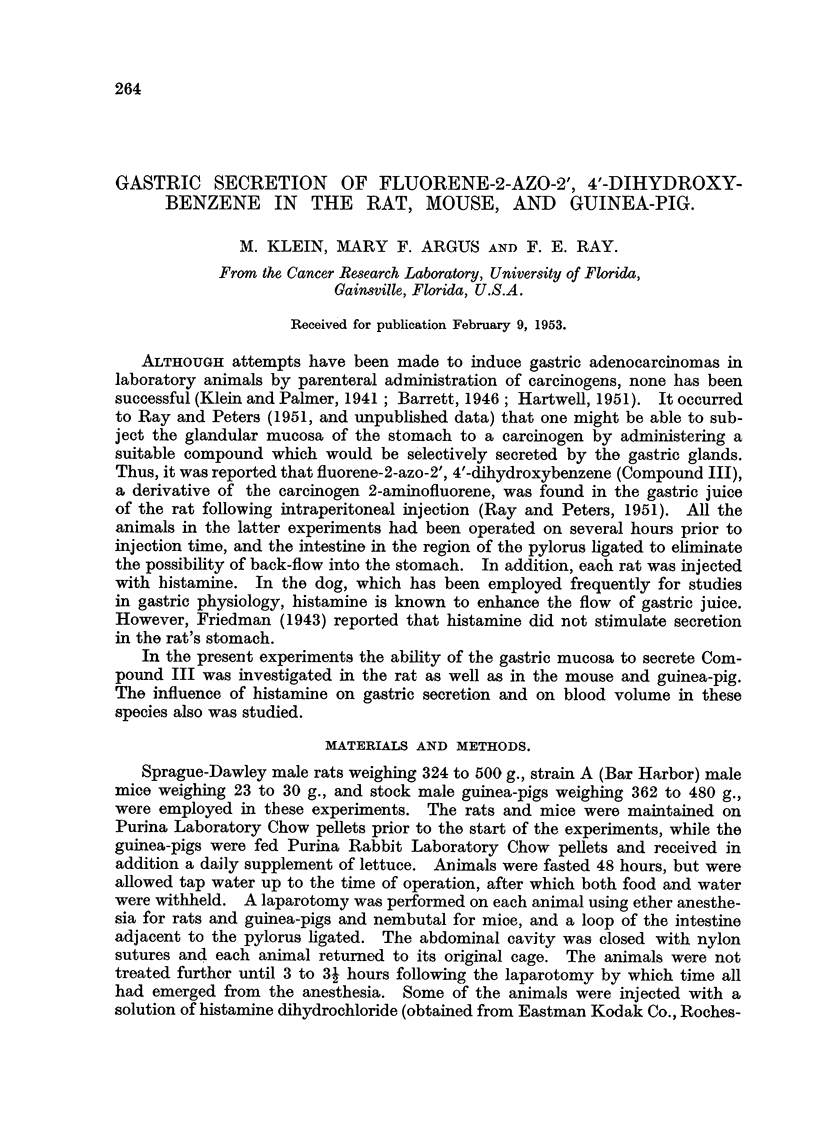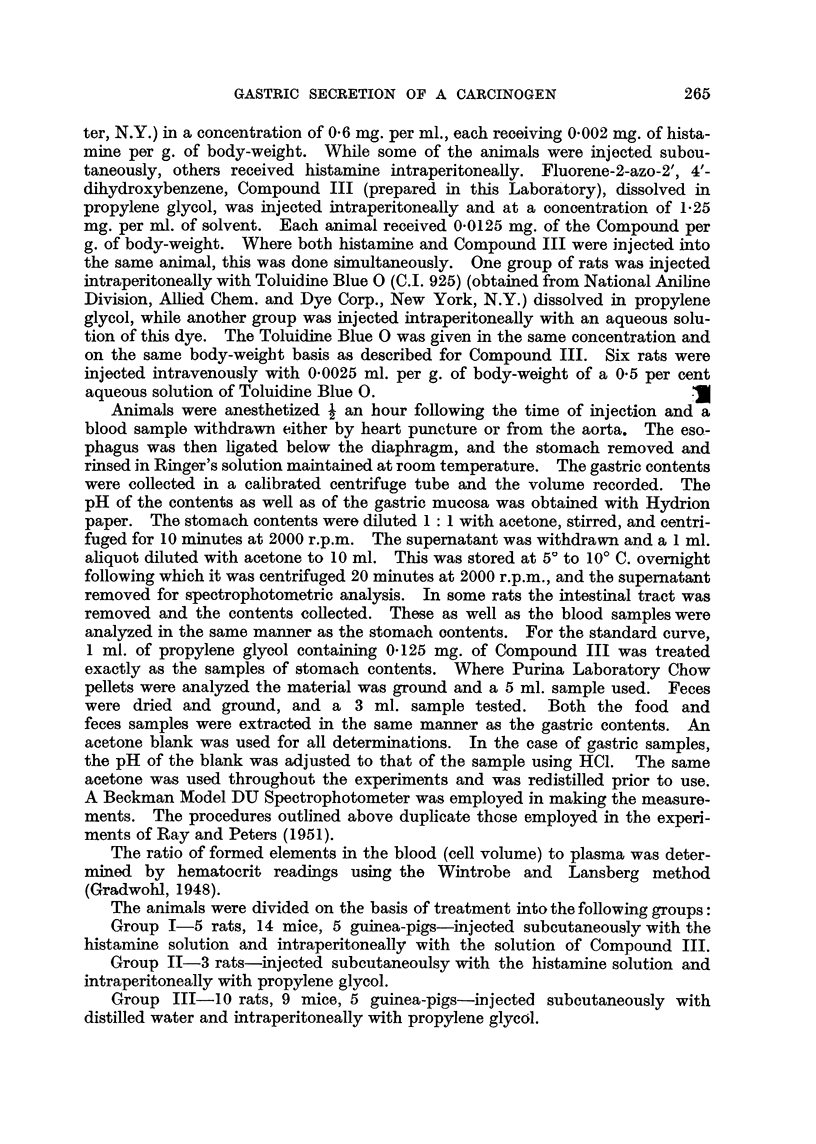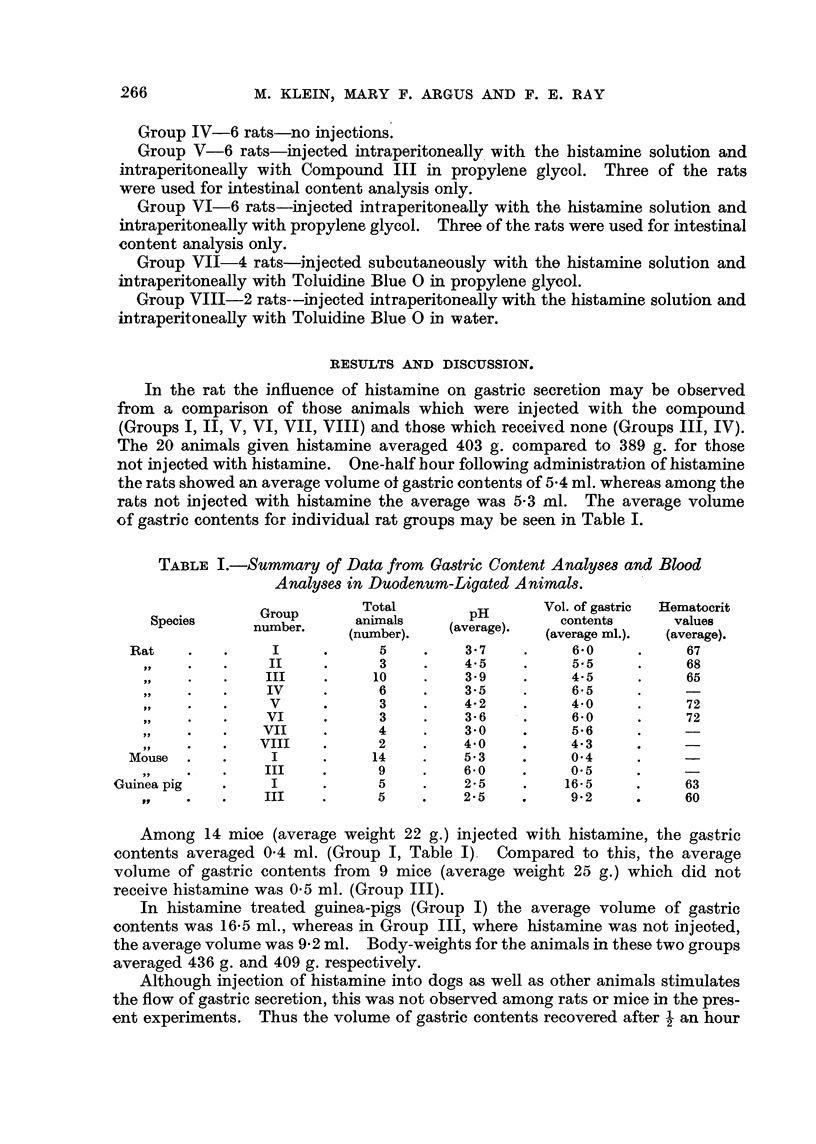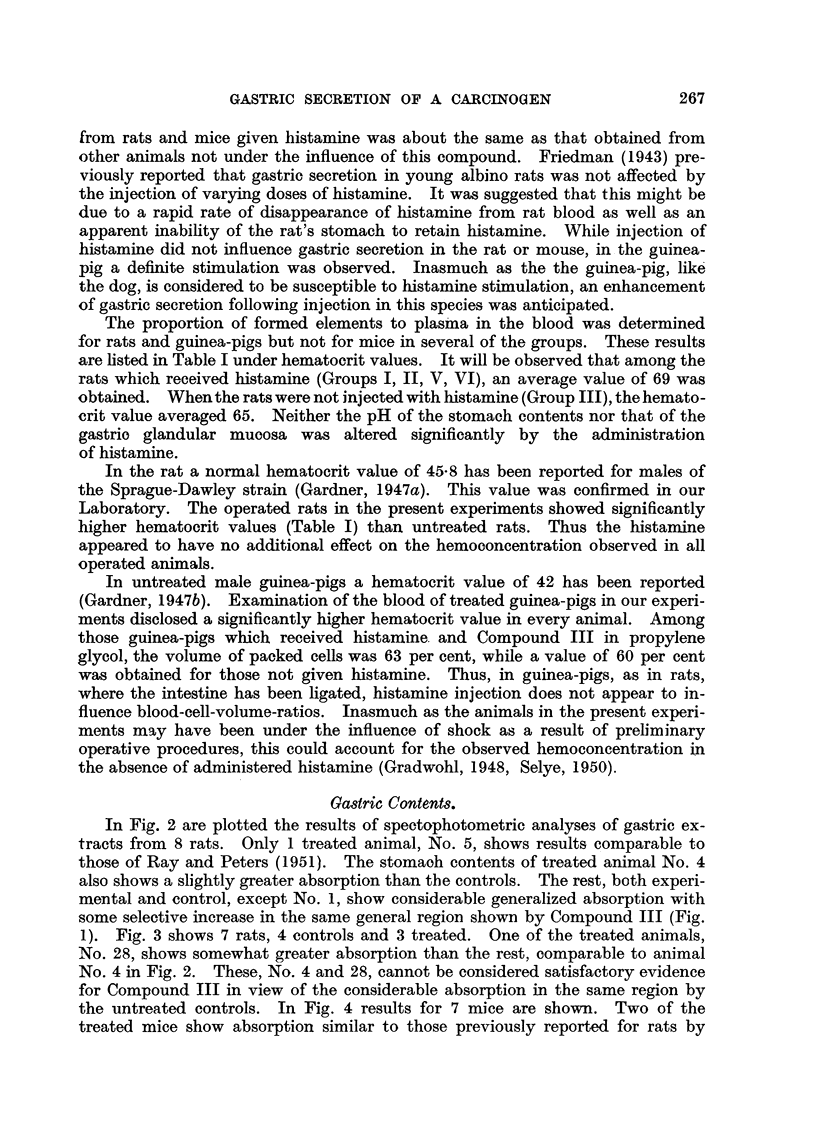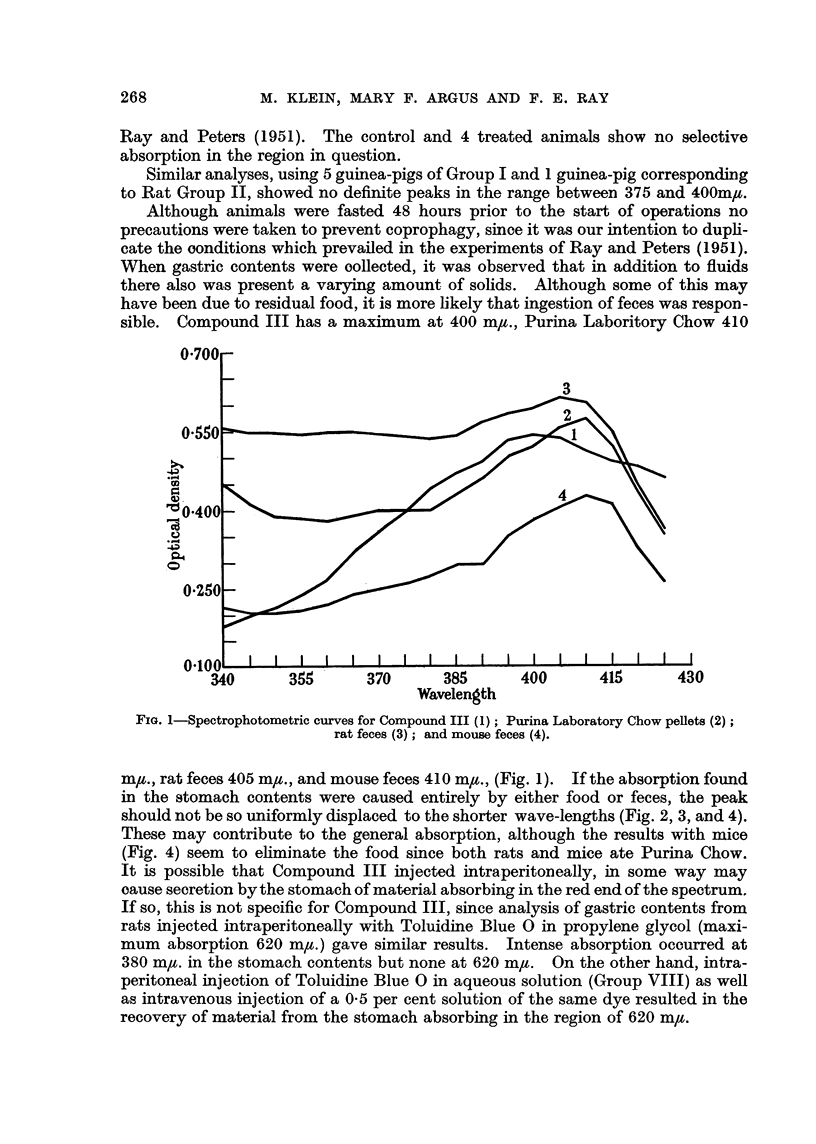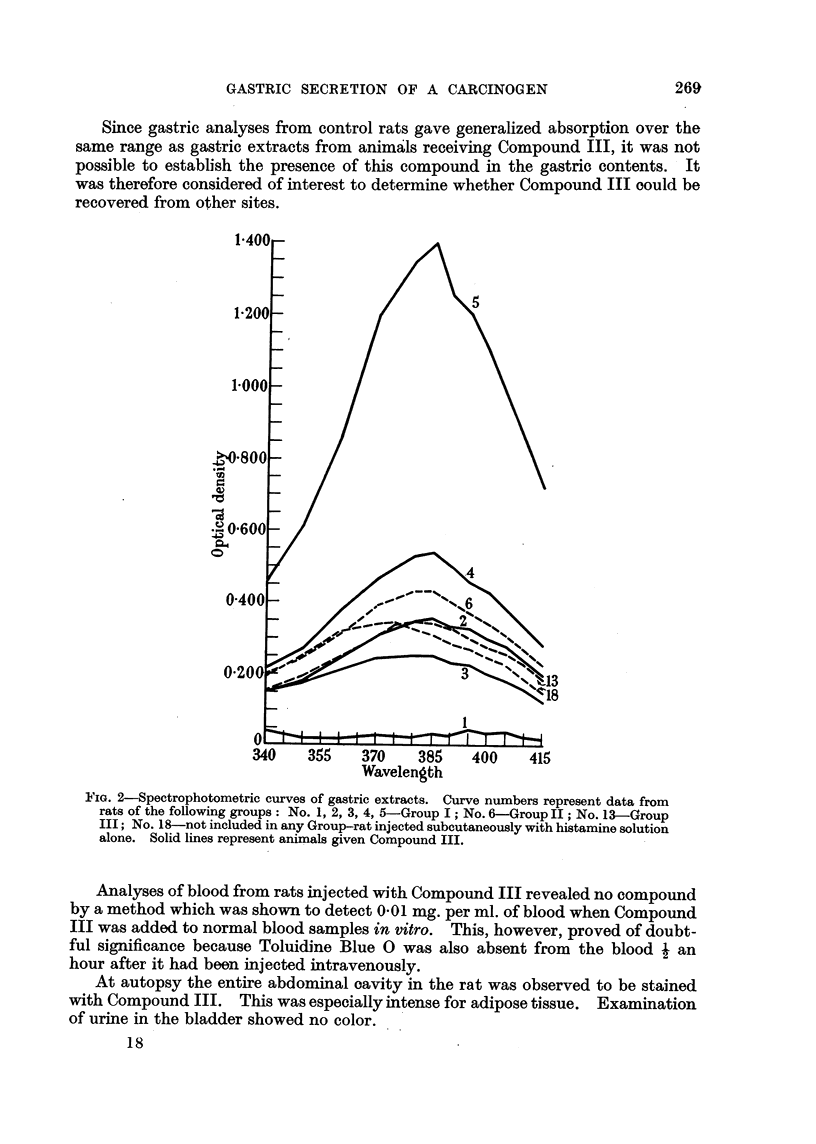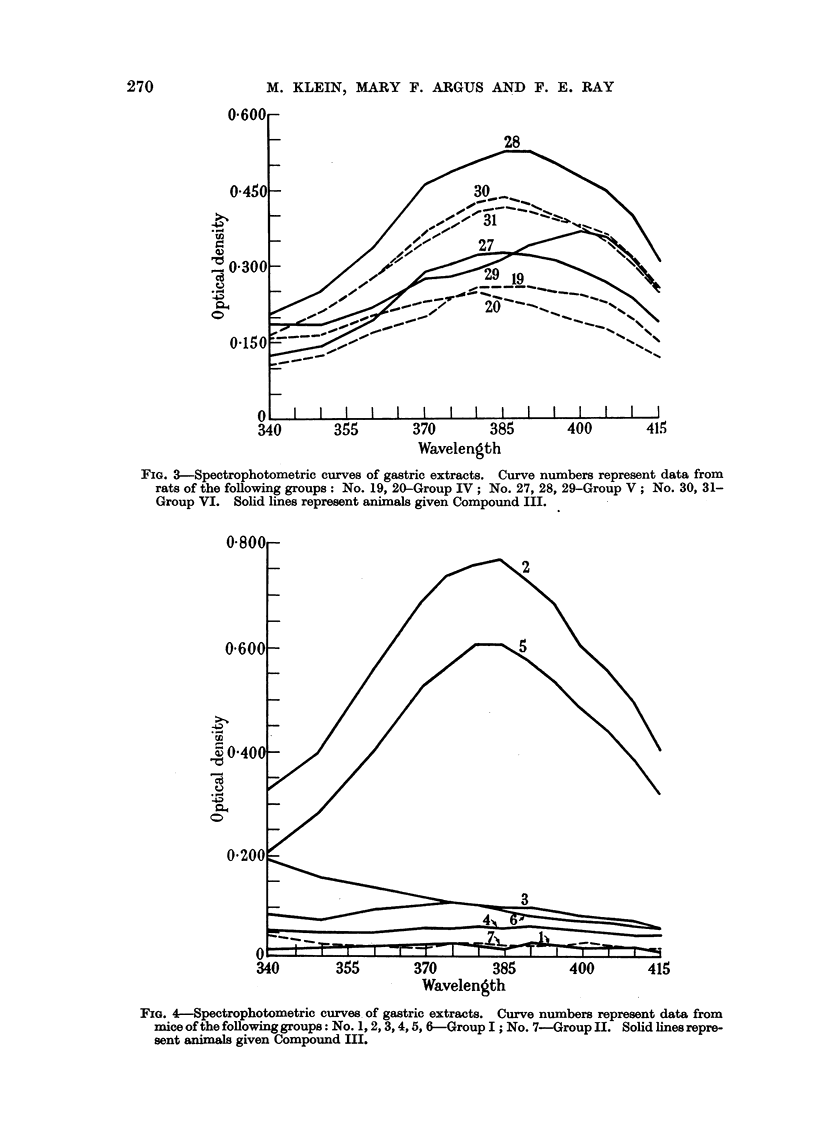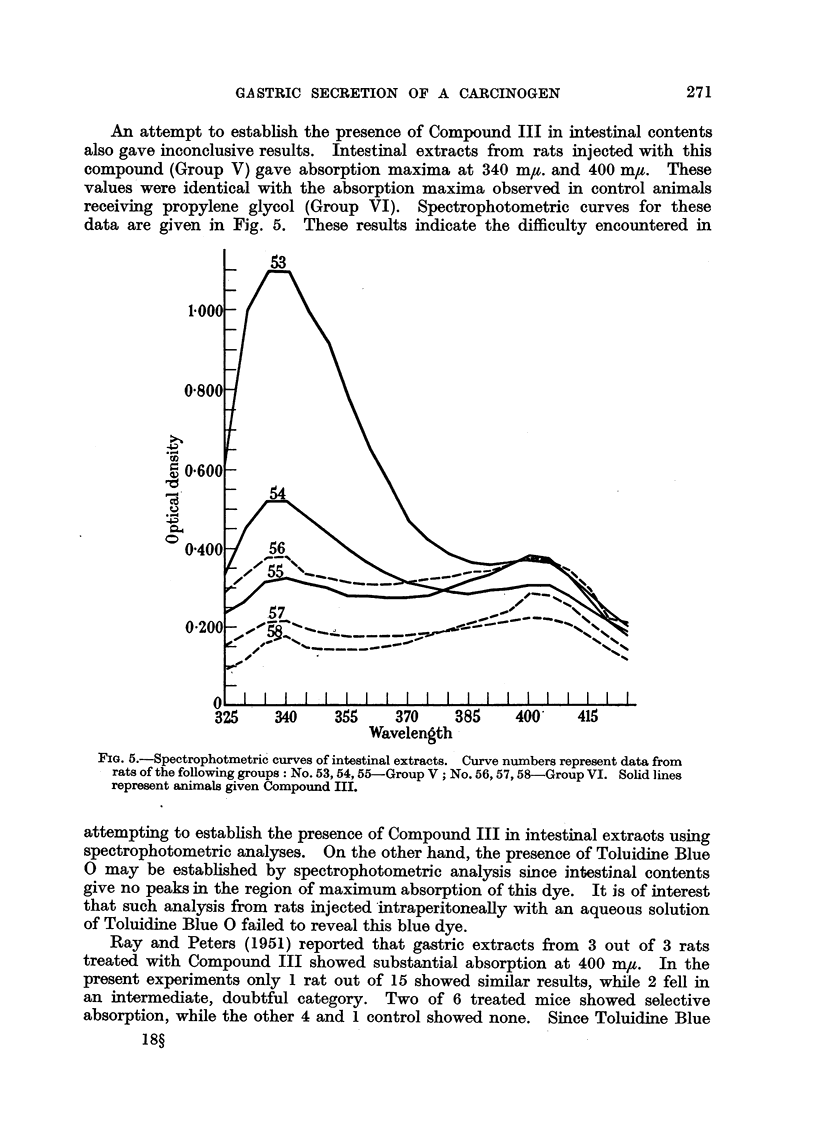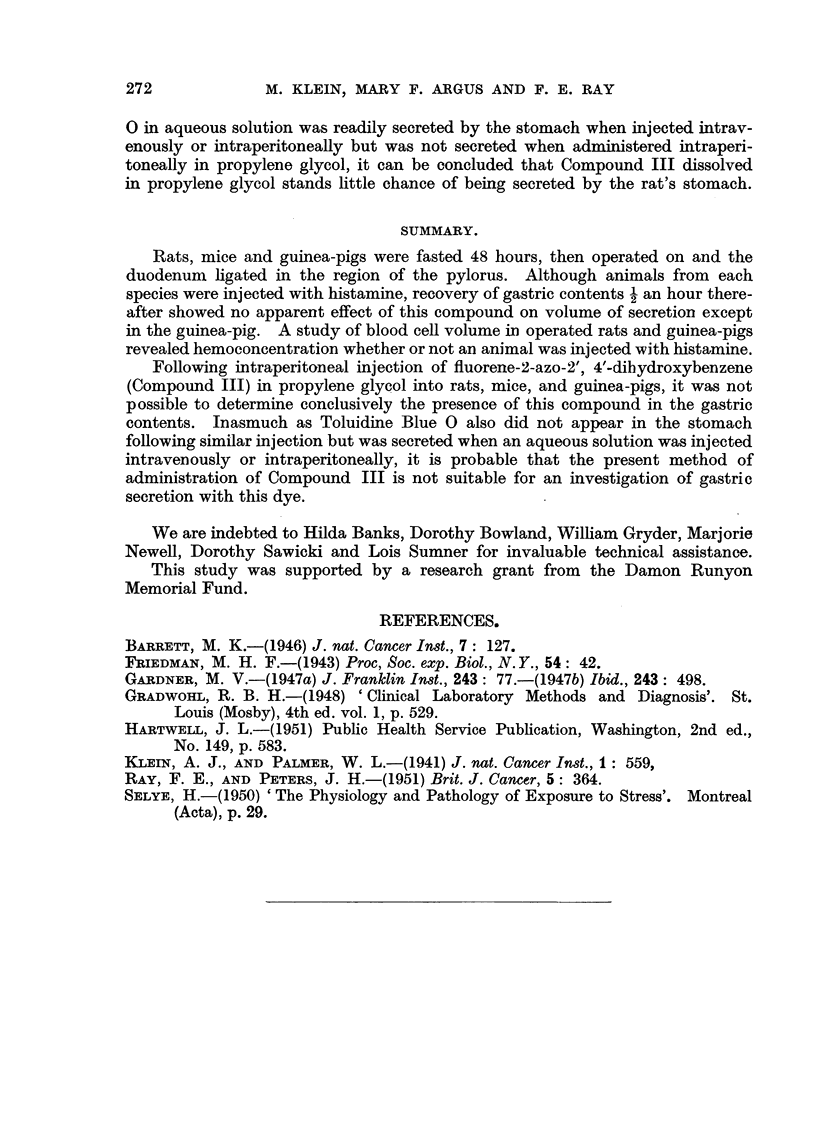# Gastric Secretion of Fluorene-2-Azo-2', 4'-Dihydroxybenzene in the Rat, Mouse, and Guinea-Pig

**DOI:** 10.1038/bjc.1953.25

**Published:** 1953-06

**Authors:** M. Klein, Mary F. Argus, F. E. Ray


### GASTRIC SECRETION OF FLUORENE-2-AZO-2′, 4′-DIHYDROXYBENZENE IN THE RAT, MOUSE, AND GUINEA-PIG.

M. KLEIN, MARY F. ARGUS AND F. E. RAY.

*From the Cancer Research Laboratory, University of Florida, Gainsville, Florida, U.S.A.*

Received for publication February 9, 1953.

ALTHOUGH attempts have been made to induce gastric adenocarcinomas in laboratory animals by parenteral administration of carcinogens, none has been successful (Klein and Palmer, 1941 ; Barrett, 1946 ; Hartwell, 1951). It occurred to Ray and Peters (1951, and unpublished data) that one might be able to subject the glandular mucosa of the stomach to a carcinogen by administering a suitable compound which would be selectively secreted by the gastric glands. Thus, it was reported that fluorene-2-azo-2′, 4′-dihydroxybenzene (Compound III), a derivative of the carcinogen 2-aminofluorene, was found in the gastric juice of the rat following intraperitoneal injection (Ray and Peters, 1951). All the animals in the latter experiments had been operated on several hours prior to injection time, and the intestine in the region of the pylorus ligated to eliminate the possibility of back-flow into the stomach. In addition, each rat was injected with histamine. In the dog, which has been employed frequently for studies in gastric physiology, histamine is known to enhance the flow of gastric juice. However, Friedman (1943) reported that histamine did not stimulate secretion in the rat's stomach.

In the present experiments the ability of the gastric mucosa to secrete Compound III was investigated in the rat as well as in the mouse and guinea-pig. The influence of histamine on gastric secretion and on blood volume in these species also was studied.

### MATERIALS AND METHODS.

Sprague-Dawley male rats weighing 324 to 500 g., strain A (Bar Harbor) male mice weighing 23 to 30 g., and stock male guinea-pigs weighing 362 to 480 g., were employed in these experiments. The rats and mice were maintained on Purina Laboratory Chow pellets prior to the start of the experiments, while the guinea-pigs were fed Purina Rabbit Laboratory Chow pellets and received in addition a daily supplement of lettuce. Animals were fasted 48 hours, but were allowed tap water up to the time of operation, after which both food and water were withheld. A laparotomy was performed on each animal using ether anesthesia for rats and guinea-pigs and nembutal for mice, and a loop of the intestine adjacent to the pylorus ligated. The abdominal cavity was closed with nylon sutures and each animal returned to its original cage. The animals were not treated further until 3 to 3½ hours following the laparotomy by which time all had emerged from the anesthesia. Some of the animals were injected with a solution of histamine dihydrochloride (obtained from Eastman Kodak Co., Rochester, N.Y.) in a concentration of 0·6 mg. per ml., each receiving 0·002 mg. of histamine per g. of body-weight. While some of the animals were injected subcutaneously, others received histamine intraperitoneally. Fluorene-2-azo-2′, 4′-dihydroxybenzene, Compound III (prepared in this Laboratory), dissolved in propylene glycol, was injected intraperitoneally and at a concentration of 1·25 mg. per ml. of solvent. Each animal received 0·0125 mg. of the Compound per g. of body-weight. Where both histamine and Compound III were injected into the same animal, this was done simultaneously. One group of rats was injected intraperitoneally with Toluidine Blue O (C.I. 925) (obtained from National Aniline Division, Allied Chem. and Dye Corp., New York, N.Y.) dissolved in propylene glycol, while another group was injected intraperitoneally with an aqueous solution of this dye. The Toluidine Blue O was given in the same concentration and on the same body-weight basis as described for Compound III. Six rats were injected intravenously with 0·0025 ml. per g. of body-weight of a 0·5 per cent aqueous solution of Toluidine Blue O.

Animals were anesthetized $\frac{1}{2}$ an hour following the time of injection and a blood sample withdrawn either by heart puncture or from the aorta. The esophagus was then ligated below the diaphragm, and the stomach removed and rinsed in Ringer's solution maintained at room temperature. The gastric contents were collected in a calibrated centrifuge tube and the volume recorded. The pH of the contents as well as of the gastric mucosa was obtained with Hydrion paper. The stomach contents were diluted 1 : 1 with acetone, stirred, and centrifuged for 10 minutes at 2000 r.p.m. The supernatant was withdrawn and a 1 ml. aliquot diluted with acetone to 10 ml. This was stored at 5° to 10° C. overnight following which it was centrifuged 20 minutes at 2000 r.p.m., and the supernatant removed for spectrophotometric analysis. In some rats the intestinal tract was removed and the contents collected. These as well as the blood samples were analyzed in the same manner as the stomach contents. For the standard curve, 1 ml. of propylene glycol containing 0·125 mg. of Compound III was treated exactly as the samples of stomach contents. Where Purina Laboratory Chow pellets were analyzed the material was ground and a 5 ml. sample used. Feces were dried and ground, and a 3 ml. sample tested. Both the food and feces samples were extracted in the same manner as the gastric contents. An acetone blank was used for all determinations. In the case of gastric samples, the pH of the blank was adjusted to that of the sample using HCl. The same acetone was used throughout the experiments and was redistilled prior to use. A Beckman Model DU Spectrophotometer was employed in making the measurements. The procedures outlined above duplicate those employed in the experiments of Ray and Peters (1951).

The ratio of formed elements in the blood (cell volume) to plasma was determined by hematocrit readings using the Wintrobe and Lansberg method (Gradwohl, 1948).

The animals were divided on the basis of treatment into the following groups:

Group I—5 rats, 14 mice, 5 guinea-pigs—injected subcutaneously with the histamine solution and intraperitoneally with the solution of Compound III.

Group II—3 rats—injected subcutaneoulsy with the histamine solution and intraperitoneally with propylene glycol.

Group III—10 rats, 9 mice, 5 guinea-pigs—injected subcutaneously with distilled water and intraperitoneally with propylene glycol.

Group IV—6 rats—no injections.

Group V—6 rats—injected intraperitoneally with the histamine solution and intraperitoneally with Compound III in propylene glycol. Three of the rats were used for intestinal content analysis only.

Group VI—6 rats—injected intraperitoneally with the histamine solution and intraperitoneally with propylene glycol. Three of the rats were used for intestinal content analysis only.

Group VII—4 rats—injected subcutaneously with the histamine solution and intraperitoneally with Toluidine Blue O in propylene glycol.

Group VIII—2 rats—injected intraperitoneally with the histamine solution and intraperitoneally with Toluidine Blue O in water.

### RESULTS AND DISCUSSION.

In the rat the influence of histamine on gastric secretion may be observed from a comparison of those animals which were injected with the compound (Groups I, II, V, VI, VII, VIII) and those which received none (Groups III, IV). The 20 animals given histamine averaged 403 g. compared to 389 g. for those not injected with histamine. One-half hour following administration of histamine the rats showed an average volume of gastric contents of 5·4 ml. whereas among the rats not injected with histamine the average was 5·3 ml. The average volume of gastric contents for individual rat groups may be seen in Table I.

TABLE I.—*Summary of Data from Gastric Content Analyses and Blood Analyses in Duodenum-Ligated Animals.*

| Species | Group number. | Total animals (number). | pH (average). | Vol. of gastric contents (average ml.). | Hematocrit values (average). |
|---|---|---|---|---|---|
| Rat | I | 5 | 3·7 | 6·0 | 67 |
| ,, | II | 3 | 4·5 | 5·5 | 68 |
| ,, | III | 10 | 3·9 | 4·5 | 65 |
| ,, | IV | 6 | 3·5 | 6·5 | — |
| ,, | V | 3 | 4·2 | 4·0 | 72 |
| ,, | VI | 3 | 3·6 | 6·0 | 72 |
| ,, | VII | 4 | 3·0 | 5·6 | — |
| ,, | VIII | 2 | 4·0 | 4·3 | — |
| Mouse | I | 14 | 5·3 | 0·4 | — |
| ,, | III | 9 | 6·0 | 0·5 | — |
| Guinea pig | I | 5 | 2·5 | 16·5 | 63 |
| ,, | III | 5 | 2·5 | 9·2 | 60 |

Among 14 mice (average weight 22 g.) injected with histamine, the gastric contents averaged 0·4 ml. (Group I, Table I). Compared to this, the average volume of gastric contents from 9 mice (average weight 25 g.) which did not receive histamine was 0·5 ml. (Group III).

In histamine treated guinea-pigs (Group I) the average volume of gastric contents was 16·5 ml., whereas in Group III, where histamine was not injected, the average volume was 9·2 ml. Body-weights for the animals in these two groups averaged 436 g. and 409 g. respectively.

Although injection of histamine into dogs as well as other animals stimulates the flow of gastric secretion, this was not observed among rats or mice in the present experiments. Thus the volume of gastric contents recovered after ½ an hour from rats and mice given histamine was about the same as that obtained from other animals not under the influence of this compound. Friedman (1943) previously reported that gastric secretion in young albino rats was not affected by the injection of varying doses of histamine. It was suggested that this might be due to a rapid rate of disappearance of histamine from rat blood as well as an apparent inability of the rat's stomach to retain histamine. While injection of histamine did not influence gastric secretion in the rat or mouse, in the guinea-pig a definite stimulation was observed. Inasmuch as the the guinea-pig, like the dog, is considered to be susceptible to histamine stimulation, an enhancement of gastric secretion following injection in this species was anticipated.

The proportion of formed elements to plasma in the blood was determined for rats and guinea-pigs but not for mice in several of the groups. These results are listed in Table I under hematocrit values. It will be observed that among the rats which received histamine (Groups I, II, V, VI), an average value of 69 was obtained. When the rats were not injected with histamine (Group III), the hematocrit value averaged 65. Neither the pH of the stomach contents nor that of the gastric glandular mucosa was altered significantly by the administration of histamine.

In the rat a normal hematocrit value of 45·8 has been reported for males of the Sprague-Dawley strain (Gardner, 1947*a*). This value was confirmed in our Laboratory. The operated rats in the present experiments showed significantly higher hematocrit values (Table I) than untreated rats. Thus the histamine appeared to have no additional effect on the hemoconcentration observed in all operated animals.

In untreated male guinea-pigs a hematocrit value of 42 has been reported (Gardner, 1947*b*). Examination of the blood of treated guinea-pigs in our experiments disclosed a significantly higher hematocrit value in every animal. Among those guinea-pigs which received histamine and Compound III in propylene glycol, the volume of packed cells was 63 per cent, while a value of 60 per cent was obtained for those not given histamine. Thus, in guinea-pigs, as in rats, where the intestine has been ligated, histamine injection does not appear to influence blood-cell-volume-ratios. Inasmuch as the animals in the present experiments may have been under the influence of shock as a result of preliminary operative procedures, this could account for the observed hemoconcentration in the absence of administered histamine (Gradwohl, 1948, Selye, 1950).

*Gastric Contents.*

In Fig. 2 are plotted the results of spectophotometric analyses of gastric extracts from 8 rats. Only 1 treated animal, No. 5, shows results comparable to those of Ray and Peters (1951). The stomach contents of treated animal No. 4 also shows a slightly greater absorption than the controls. The rest, both experimental and control, except No. 1, show considerable generalized absorption with some selective increase in the same general region shown by Compound III (Fig. 1). Fig. 3 shows 7 rats, 4 controls and 3 treated. One of the treated animals, No. 28, shows somewhat greater absorption than the rest, comparable to animal No. 4 in Fig. 2. These, No. 4 and 28, cannot be considered satisfactory evidence for Compound III in view of the considerable absorption in the same region by the untreated controls. In Fig. 4 results for 7 mice are shown. Two of the treated mice show absorption similar to those previously reported for rats by

Ray and Peters (1951). The control and 4 treated animals show no selective absorption in the region in question.

Similar analyses, using 5 guinea-pigs of Group I and 1 guinea-pig corresponding to Rat Group II, showed no definite peaks in the range between 375 and 400m$\mu$.

Although animals were fasted 48 hours prior to the start of operations no precautions were taken to prevent coprophagy, since it was our intention to duplicate the conditions which prevailed in the experiments of Ray and Peters (1951). When gastric contents were collected, it was observed that in addition to fluids there also was present a varying amount of solids. Although some of this may have been due to residual food, it is more likely that ingestion of feces was responsible. Compound III has a maximum at 400 m$\mu$., Purina Laboritory Chow 410

FIG. 1—Spectrophotometric curves for Compound III (1) ; Purina Laboratory Chow pellets (2) ; rat feces (3) ; and mouse feces (4).

m$\mu$., rat feces 405 m$\mu$., and mouse feces 410 m$\mu$., (Fig. 1). If the absorption found in the stomach contents were caused entirely by either food or feces, the peak should not be so uniformly displaced to the shorter wave-lengths (Fig. 2, 3, and 4). These may contribute to the general absorption, although the results with mice (Fig. 4) seem to eliminate the food since both rats and mice ate Purina Chow. It is possible that Compound III injected intraperitoneally, in some way may cause secretion by the stomach of material absorbing in the red end of the spectrum. If so, this is not specific for Compound III, since analysis of gastric contents from rats injected intraperitoneally with Toluidine Blue O in propylene glycol (maximum absorption 620 m$\mu$.) gave similar results. Intense absorption occurred at 380 m$\mu$. in the stomach contents but none at 620 m$\mu$. On the other hand, intraperitoneal injection of Toluidine Blue O in aqueous solution (Group VIII) as well as intravenous injection of a 0·5 per cent solution of the same dye resulted in the recovery of material from the stomach absorbing in the region of 620 m$\mu$.

Since gastric analyses from control rats gave generalized absorption over the same range as gastric extracts from animals receiving Compound III, it was not possible to establish the presence of this compound in the gastric contents. It was therefore considered of interest to determine whether Compound III could be recovered from other sites.

FIG. 2—Spectrophotometric curves of gastric extracts. Curve numbers represent data from rats of the following groups : No. 1, 2, 3, 4, 5—Group I ; No. 6—Group II ; No. 13—Group III ; No. 18—not included in any Group–rat injected subcutaneously with histamine solution alone. Solid lines represent animals given Compound III.

Analyses of blood from rats injected with Compound III revealed no compound by a method which was shown to detect 0·01 mg. per ml. of blood when Compound III was added to normal blood samples *in vitro*. This, however, proved of doubtful significance because Toluidine Blue O was also absent from the blood ½ an hour after it had been injected intravenously.

At autopsy the entire abdominal cavity in the rat was observed to be stained with Compound III. This was especially intense for adipose tissue. Examination of urine in the bladder showed no color.

FIG. 3—Spectrophotometric curves of gastric extracts. Curve numbers represent data from rats of the following groups : No. 19, 20–Group IV ; No. 27, 28, 29–Group V ; No. 30, 31–Group VI. Solid lines represent animals given Compound III.

FIG. 4—Spectrophotometric curves of gastric extracts. Curve numbers represent data from mice of the following groups : No. 1, 2, 3, 4, 5, 6—Group I ; No. 7—Group II. Solid lines represent animals given Compound III.

An attempt to establish the presence of Compound III in intestinal contents also gave inconclusive results. Intestinal extracts from rats injected with this compound (Group V) gave absorption maxima at 340 m$\mu$. and 400 m$\mu$. These values were identical with the absorption maxima observed in control animals receiving propylene glycol (Group VI). Spectrophotometric curves for these data are given in Fig. 5. These results indicate the difficulty encountered in

FIG. 5.—Spectrophotmetric curves of intestinal extracts. Curve numbers represent data from rats of the following groups : No. 53, 54, 55—Group V ; No. 56, 57, 58—Group VI. Solid lines represent animals given Compound III.

attempting to establish the presence of Compound III in intestinal extracts using spectrophotometric analyses. On the other hand, the presence of Toluidine Blue O may be established by spectrophotometric analysis since intestinal contents give no peaks in the region of maximum absorption of this dye. It is of interest that such analysis from rats injected intraperitoneally with an aqueous solution of Toluidine Blue O failed to reveal this blue dye.

Ray and Peters (1951) reported that gastric extracts from 3 out of 3 rats treated with Compound III showed substantial absorption at 400 m$\mu$. In the present experiments only 1 rat out of 15 showed similar results, while 2 fell in an intermediate, doubtful category. Two of 6 treated mice showed selective absorption, while the other 4 and 1 control showed none. Since Toluidine Blue

O in aqueous solution was readily secreted by the stomach when injected intravenously or intraperitoneally but was not secreted when administered intraperitoneally in propylene glycol, it can be concluded that Compound III dissolved in propylene glycol stands little chance of being secreted by the rat's stomach.

SUMMARY.

Rats, mice and guinea-pigs were fasted 48 hours, then operated on and the duodenum ligated in the region of the pylorus. Although animals from each species were injected with histamine, recovery of gastric contents $\frac{1}{2}$ an hour thereafter showed no apparent effect of this compound on volume of secretion except in the guinea-pig. A study of blood cell volume in operated rats and guinea-pigs revealed hemoconcentration whether or not an animal was injected with histamine.

Following intraperitoneal injection of fluorene-2-azo-2′, 4′-dihydroxybenzene (Compound III) in propylene glycol into rats, mice, and guinea-pigs, it was not possible to determine conclusively the presence of this compound in the gastric contents. Inasmuch as Toluidine Blue O also did not appear in the stomach following similar injection but was secreted when an aqueous solution was injected intravenously or intraperitoneally, it is probable that the present method of administration of Compound III is not suitable for an investigation of gastric secretion with this dye.

We are indebted to Hilda Banks, Dorothy Bowland, William Gryder, Marjorie Newell, Dorothy Sawicki and Lois Sumner for invaluable technical assistance.

This study was supported by a research grant from the Damon Runyon Memorial Fund.

### REFERENCES.

BARRETT, M. K.—(1946) *J. nat. Cancer Inst.*, **7** : 127.

FRIEDMAN, M. H. F.—(1943) *Proc, Soc. exp. Biol., N.Y.*, **54** : 42.

GARDNER, M. V.—(1947*a*) *J. Franklin Inst.*, **243** : 77.—(1947*b*) *Ibid.*, **243** : 498.

GRADWOHL, R. B. H.—(1948) 'Clinical Laboratory Methods and Diagnosis'. St. Louis (Mosby), 4th ed. vol. 1, p. 529.

HARTWELL, J. L.—(1951) Public Health Service Publication, Washington, 2nd ed., No. 149, p. 583.

KLEIN, A. J., AND PALMER, W. L.—(1941) *J. nat. Cancer Inst.*, **1** : 559,

RAY, F. E., AND PETERS, J. H.—(1951) *Brit. J. Cancer*, **5** : 364.

SELYE, H.—(1950) 'The Physiology and Pathology of Exposure to Stress'. Montreal (Acta), p. 29.